# Spatiotemporal patterns and optimization of dominant and recessive morphology of cultivated land use in the Ili River Valley

**DOI:** 10.1016/j.isci.2026.116221

**Published:** 2026-06-11

**Authors:** Chunmei Zhou, Lihua Fu, Hui Xiang

**Affiliations:** 1 Institute of Resources and Ecology, Yili Normal University, Yining 835000, China; 2 College of Resources and Environment, Yili Normal University, Yining 835000, China; 3 College of Geography and Tourism, Hunan University of Arts and Science, Changde, Hunan 415000, China

**Keywords:** Environmental science, Environmental issues, Land use

## Abstract

In recent years, the rapid expansion and functional degradation of cultivated land have intensified the pressure on this crucial resource, and the protection of cultivated land needs urgent attention. Based on an integrated view of dominant and recessive, combined with landscape index selection, spatial mapping method, kernel density estimation, and coupling coordination degree, this study examines the spatiotemporal changes in cultivated land in the Ili River Valley from 2005 to 2020 and proposes optimization strategies. Key findings include: (1) The dominant morphology of cultivated land use shows a slow growth trend, and the recessive morphology (total function) exhibits a faster growth rate than that of the dominant morphology. (2) The coupling coordination has gradually shifted from barely coordinated and primary coordination to intermediate coordination. (3) The region is divided into three primary zones and six secondary zones, and this study proposes targeted differentiated development strategies. It is helpful to the sustainable utilization of cultivated land and the rational planning of land space in arid oasis regions.

## Highlights

• Two-dimensional index of dominant and recessive cultivated land is constructed
• Dominant morphology grows slowly while recessive morphology grows faster
• Coupling coordination shifts from barely and primary to intermediate level
• Three primary and six secondary zones are identified with differentiated strategies

## Graphical abstract

**Figure:**
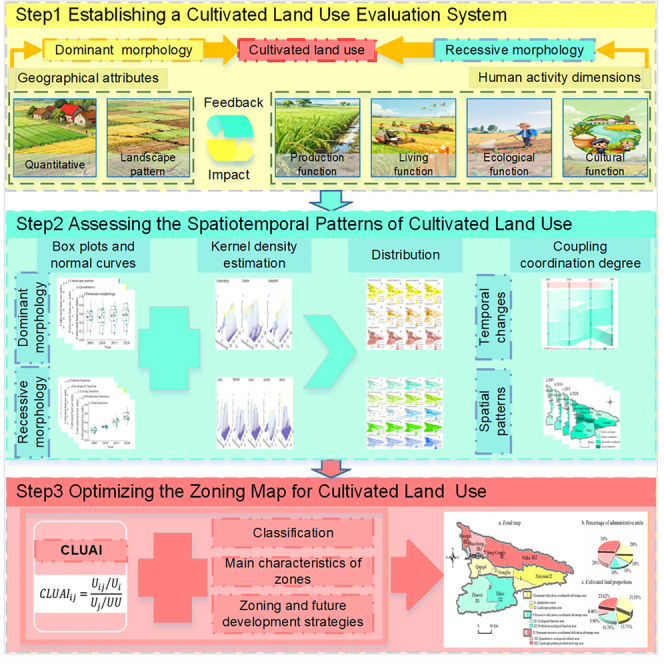

## Introduction

Cultivated land is the key factor to achieve grain production and socioeconomic development, and it is an important carrier to protect ecological security and preserve agricultural civilization.^1^ However, there are numerous internal and external challenges in China’s food security, and the utilization of cultivated land is under various pressures. To begin with, the superposition of multiple factors such as global political conflicts, trade disputes, worldwide public health events, climate change and local famine has intensified the uncertainty of external food supply.^2^ Moreover, domestic factors such as abandoned farmland, deteriorating ecosystems and overexploitation have disrupted agricultural practices, leading to the decline of cultivated land utilization, spatial encroachment and functional degradation,^3^ which poses significant challenges to the sustainable utilization of cultivated land and the high-quality agricultural development.^4^ To effectively address these issues, the 2025 Central Document No. 1 mandates “strengthening cultivated land protection and quality improvement,” emphasizing “strict control of total cultivated land,” and “promoting the safe use of contaminated cultivated land.” Against this backdrop, it is of great practical significance to explore the evolution of cultivated land use and zoning optimization strategies in alleviating the pressure of cultivated land use, ensuring food security, and promoting healthy and sustainable economic development.^5^

In recent years, with the deepening interpretation and excavation of cultivated land use by scholars, research findings have gradually become more comprehensive and refined. Among them, the research on cultivated land use can be divided into two categories: theoretical and empirical. Theoretical research mainly focuses on the concept construction and expansion, theoretical development, and innovation of cultivated land use. A clear understanding and accurate interpretation of the concept of cultivated land use is the fundamental prerequisite for studying the dominant and recessive morphologies of cultivated land use. As a fundamental factor supporting regional development, the overall pattern and utilization the overall pattern and utilization forms of cultivated land resources are highly susceptible to change under the impetus of economic development and innovation. It has mainly undergone a paradigm shift from quantity control to quality-ecology synergy. Inspired by the forest transition hypothesis, Grainger pioneered the theory of land use transition, initiating the study of observable dominant morphology such as quantity, spatial structure and planting pattern.^6^ Subsequently, Lambin and Meyfroidt constructed a theoretical framework for land use transformation based on the dual perspectives of social-ecological feedback and socio-economic change.^7^ Long and Li introduced this theory into China,^8^ and subsequently further expanded the dominant and recessive morphologies dual analysis framework.^9^ The introduction of this theory provides a theoretical basis for understanding the evolution of the intrinsic characteristics of cultivated land. The 2025 Central Document No. 1 further emphasized the strengthening of cultivated land protection and quality improvement. The “trinity” protection of cultivated land quantity, quality, and ecology has become the main direction of cultivated land utilization optimization practice in China.^10^ At this point, the connotation of cultivated land use has been relatively well defined. It usually refers to the spatial patterns, structural distribution, allocation characteristics and functional forms exhibited by the human development of cultivated land within specific periods of socioeconomic development.^11^ Scholars generally believe that cultivated land use manifests in two types: dominant and recessive morphology.^12^ Dominant morphology of cultivated land use manifests in quantitative aspects and landscape features, while recessive morphology of cultivated land use comes from different functional forms caused by different management models and organizational methods.^13^ The empirical research on cultivated land use mainly focuses on the selection of evaluation indicators, research methods, research areas and optimization strategies. Existing research primarily focuses on exploring either dominant or recessive morphology of land use, with relatively few studies quantitatively analyzing two-dimensional morphologies.^14^ There are abundant evaluation indicators, the dominant morphology of cultivated land use primarily encompasses area, reclamation rate^15^ and spatial distribution patterns,^16^ while recessive morphology of cultivated land use focuses on analyzing three functional categories: production, ecological and social,^17^ lacking an interpretation of cultural functions from the perspective of agricultural civilization inheritance. Research methods have become increasingly diversified, with spatial mapping method,^18^ entropy method,^19^ composite indicator weighting^20^ and spatial econometric model^21^ being widely applied to explore the spatiotemporal evolution characteristics of cultivated land use. Among them, the spatial mapping method has simple calculation logic and strong operability, enabling intuitive visualization of overall spatial characteristics and local spatial differences.^22^ It is the mainstream model to reveal the spatial heterogeneity of cultivated land use.^19^ However, the exploration of its temporal variation patterns remains constrained by static descriptions, and dynamic and visualizable techniques are still under exploration.^23^ Kernel density estimation can dynamically and intuitively reveal the temporal evolution characteristics and developmental trends of phenomena from multiple perspectives.^24^^,^^25^ It effectively addresses the aforementioned shortcomings, but few studies have applied it to the research on cultivated land use change.^25^ The coupling coordination degree model effectively overcomes the limitations of previous studies that only evaluated a single system, and can comprehensively reflect the coordinated development status of multiple systems.^26^ Research areas continue to expand, and the existing research has conducted extensive theoretical and empirical exploration on the Yangtze River Basin,^27^ the Huang-Huai-Hai region,^28^ typical mountainous areas^29^ and plains.^30^ It is primarily focused on the central and eastern China, and fewer research involving the arid oasis regions in Northwest China.^31^ The arid oasis regions of Northwest China, situated in the heart of the Eurasian continent, serve as a vital gateway for China’s opening-up to the west. It represents the core area and key field for current and future territorial development in China.^32^ However, the ecosystems in the arid oasis regions of Northwest China exhibit poor stability and weak resistance to disturbances,^33^ which is a typical ecologically sensitive and ecologically fragile regions.^34^ Coupled with the constraints of population and socioeconomic development, there is great uncertainty in the development of regional cultivated land. Opportunities and challenges coexist in the development of cultivated land resources, and the protection of cultivated land needs urgent attention.^35^ The optimization strategies have become increasingly refined. Scholars usually quantify land use function values through biophysical measurement and economic evaluation methods to analyze the spatial functional zoning of the “ecological-production-living” in rural areas.^36^ The coupling coordination degree and K-means clustering methods were employed to identify the coordination level and partition types.^10^ Therefore, the theoretical research on cultivated land use provides an important literature research foundation for this study. However, a previous study has clearly pointed out that the research on cultivated land use particularly requires conducting empirical studies in ecologically fragile areas in order to verify and improve the theories.^37^

Given this, the Ili River Valley is selected as its case study area, which integrates the arid oasis region, agricultural and pastoral production zones, ecological civilization demonstration areas, and ethnic cultural exchange zones in Northwest China. Based on the comprehensive perspectives of dominant and recessive morphology, combined with landscape index selection, spatial mapping method, kernel density estimation, and coupling coordination degree, this study analyzes the spatiotemporal evolution of cultivated land use from 2005 to 2020 and proposes optimization strategies for zoning and differentiated development. This study answers three key questions: (1) What patterns emerge in the temporal evolution of dominant and recessive morphology of cultivated land use in the Ili River Valley? (2) What are the differences in spatial patterns? (3) What are the future utilization directions for cultivated land in different regions? This study aims to provide a scientific basis for the conservation and utilization of cultivated land resources and the coordinated development of human-land relations in the arid oasis regions, thereby serving regional agricultural sustainability and comprehensive rural revitalization.

## Results

### Spatiotemporal patterns of the dominant morphology of cultivated land use

#### Temporal evolution of the dominant morphology of cultivated land use

**Figure 1:**
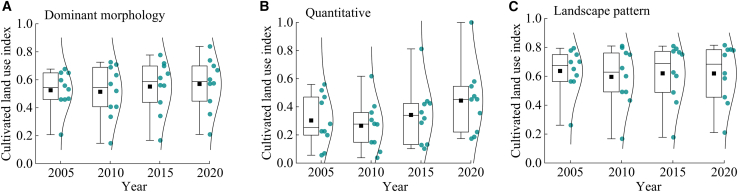
Boxplot and normal curve plot of dominant morphology of cultivated land use in the Ili River Valley, 2005-2020

The temporal evolution characteristics of the dominant morphology of cultivated land use in the Ili River Valley from 2005 to 2020 were analyzed using boxplots and normal curves (Figure 1). As shown in the figure, the dominant morphology of the cultivated land use index increased from 0.5251 in 2005 to 0.5701 in 2020, with an increase rate of 8.57% and an average annual change rate of 0.57%, showing a slow growth trend. Among them, the quantitative characteristic index increased from 0.3030 in 2005 to 0.4444 in 2020, with an increase rate of 31.82% and an average annual change rate of 2.12%, reflecting the slow growth of quantity. The landscape pattern index decreased from 0.6366 to 0.6194, with a decrease of −2.70% and an average annual change rate of −0.18%, indicating that the landscape pattern declined steadily. The possible reason is that since 2005, the population of the Ili River Valley has continued to grow, and the cultivated land reclamation rate has increased from 0.0822 in 2005 to 0.1420 in 2020. This has enhanced the continuous improvement of the utilization intensity of existing cultivated land resources, thereby promoting the dominant morphology of cultivated land use and the improvement of quantitative characteristics. However, the strictest cultivated land protection policy in China has limited the blind development and quantitative expansion of cultivated land to some extent. Meanwhile, socio-economic activities such as urban construction and industrial mining development have led to the diversification, fragmentation, and discretization of cultivated land use around the city, which makes the landscape pattern index decrease slightly.

**Figure 2:**
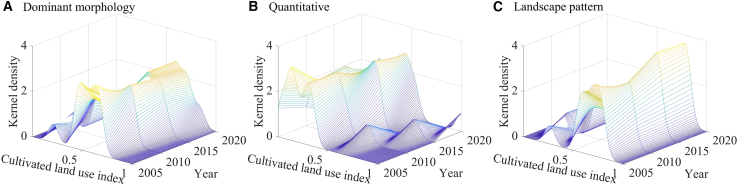
3D kernel density plot of dominant morphology of cultivated land use in the Ili River Valley, 2005-2020

The dynamic evolution characteristics of the dominant morphology of cultivated land use in the region from 2005 to 2020 were characterized using kernel density estimation (Figure 2). From the distribution position, there is no obvious left or right shift tendency in the center of the kernel density curve of the dominant morphology of cultivated land use and its secondary indicators (quantitative and landscape pattern). It indicates that the dominant morphology of cultivated land use did not fluctuate greatly during the study period, maintaining a relatively stable development trend. From the distribution pattern, the height of the core density curve of the dominant morphology of cultivated land use and quantitative characteristics in the Ili River Valley decreases, and its width widens. It shows that the dispersion degree increases and the absolute difference is relatively unstable. However, the height of the main peak of the landscape pattern kernel density curve decreased slightly, then rose significantly, and finally stabilized, and the width also changed obviously. At the same time, some regions exhibit greater height and narrower width, reflecting significant absolute differences in the cultivated land landscape. From the polarization phenomenon, the kernel density curves for both dominant morphologies of cultivated land use and quantitative characteristics in the Ili River Valley exhibit unimodal distributions, with signs of fluctuation at the end of the curve from 2005 to 2015. It shows that there is local polarization, but the fluctuation slows down from 2015 to 2020, with the single-peak polarization phenomenon becoming increasingly prominent. The kernel density curve of landscape pattern exhibited significant local differences from 2005 to 2010, gradually developed to a single peak from 2010 to 2015, and the peak value significantly decreased from 2015 to 2020. It exhibits an evolutionary trend of “dispersion (2005–2010), convergence (2010–2015), and diffusion (2015–2020).”

#### Spatial patterns of the dominant morphology of cultivated land use

**Figure 3:**
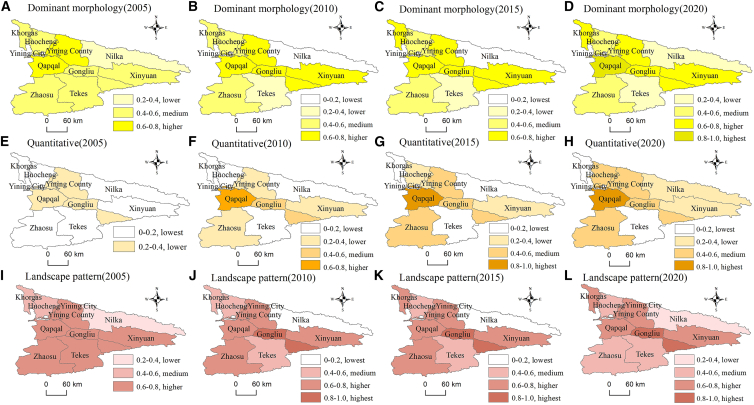
Spatial patterns of dominant morphology of cultivated land use in the Ili River Valley Note: the dominant morphology of cultivated land use is divided into five levels, lowest (0–0.2), lower (0.2–0.4), medium (0.4–0.6), higher (0.6–0.8), and highest (0.8–1), and the same below.

The distribution pattern of the dominant morphology of cultivated land use in the study area from 2005 to 2020 is shown in Figure 3. During the study period, the dominant morphology of cultivated land use exhibited spatial heterogeneity. In 2005, the medium-value regions were mainly concentrated in the form of clusters in the northwest and south. Yining City, Yining County, and Qapqal in the middle were higher value regions, while Nilka in the northeast was the only region with a lower value (Figure 3A). In particular, the spatial patterns of dominant morphology of cultivated land use in 2010 and 2015 were basically consistent (Figures 3B and 3C). Yining County, Qapqal, Gongliu, and Xinyuan in the central part of the river valley are regions with higher value, exhibiting a spatial characteristic of “high in the valley and low on both sides.” In 2020, only Qapqal in the west rose to the highest value region (Figure 3D). The lower and medium value regions were scattered on both sides of the river valley, covering a total of five administrative units, accounting for 50%.

From 2005 to 2020, the spatial difference of quantitative characteristics of cultivated land in the Ili River Valley was significant. In 2005, lower values exhibited a strip-like trend from northwest to southeast, including four administrative units: Yining City, Yining County, Qapqal, and Gongliu (Figure 3E). The rest are all at the lowest levels. In 2010 and 2015, the spatial pattern of quantitative characteristics of cultivated land gradually stabilized (Figures 3F and 3G). Its core area is located in the west (Qapqal), and the level of quantitative characteristics of most administrative units has increased. In 2020, the quantitative characteristics of cultivated land show a spatial pattern of “highest value is locally prominent, and medium value is generally distributed” (Figure 3H). Among them, the highest value region is still located in the west, and the medium value region is roughly northwest-southeast. Lower value regions are distributed in the eastern region in blocks, and the lowest value regions are scattered in the northwest and southwest.

From 2005 to 2020, the landscape pattern of cultivated land in the Ili River Valley exhibited a spatial pattern similar to that of dominant morphology, with higher values on both sides of the river valley and lower values in the middle. In 2005, the distribution pattern of the region was high in the south and low in the north, with higher value regions mainly concentrated in the south (Figure 3I). There was a total of seven administrative units, accounting for 70%. In 2010 and 2015, the spatial distribution pattern of the cultivated land landscape pattern further stabilized (Figures 3J and 3K). Gongliu in the south stood out as the only highest-value region, while medium-value regions were clustered in the central part. Lower-value and medium-value regions were scattered in the northwest (Khorgas and Huocheng), the south (Tekes), and the northeast (Nilka). In 2020, the low-lying regions on both sides of the valley were obvious, and most regions maintained their original grades, with only a few regions experiencing changes (Zhaosu in the southwest and Nilka in the northeast) (Figure 3L).

### Spatiotemporal patterns of recessive morphology of cultivated land use

#### Temporal evolution of recessive morphology of cultivated land use

**Figure 4:**
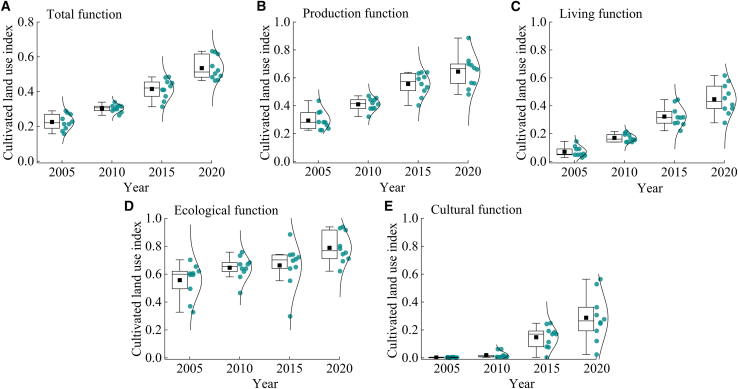
Boxplot and normal curve plot of recessive morphology of cultivated land use in the Ili River Valley, 2005-2020

The temporal evolution characteristics of the recessive morphology of cultivated land use in the Ili River Valley from 2005 to 2020 are shown in Figure 4. The recessive morphology of the cultivated land use index (total function) increased from 0.2257 in 2005 to 0.5353 in 2020, with an increase rate of 137.17% and an average annual change rate of 9.14%, which was more obvious than that of the dominant morphology of cultivated land use. Meanwhile, all sub-systems have different degrees of growth. The cultural function index surged from 0.0027 in 2005 to 0.2875 in 2020, with an increase rate of 10548.15% and an average annual change rate of 703.21%, showing an explosive growth trend. The living function index rose from 0.0707 to 0.4466, with an increase rate of 531.68% and an average annual change rate of 35.45%, which is characterized by rapid growth. The production function index increased from 0.2938 to 0.6450, representing a growth rate of 119.54%. The average annual growth rate was 7.97%, indicating a moderate growth rate. The ecological function index rose from 0.5569 to 0.7889, representing a growth rate of 41.66%. The annual average change rate was 2.78%, marking the slowest growth rate among all functions. The above data show that relying on the advantages of the Silk Road Economic Belt, the agricultural cultural industry in the Ili River Valley is developing rapidly and may become a key engine for the high-quality utilization of regional cultivated land. Concurrently, with the construction of agricultural infrastructure and the matching of preferential policies, the farmer income intensity per unit of cultivated land increased from 18,655.98 yuan/hm^2^ in 2005 to 53,157.29 yuan/hm^2^ in 2020. This has promoted the increase of agricultural production and farmers' income, leading to an enhancement in both production and living functions. In addition, the gradual improvement of ecological function indicates that the construction of ecological civilization has become the core policy of cultivated land resources management in the Ili River Valley, and cultivated land use pays more attention to quality improvement than blind expansion.

**Figure 5:**
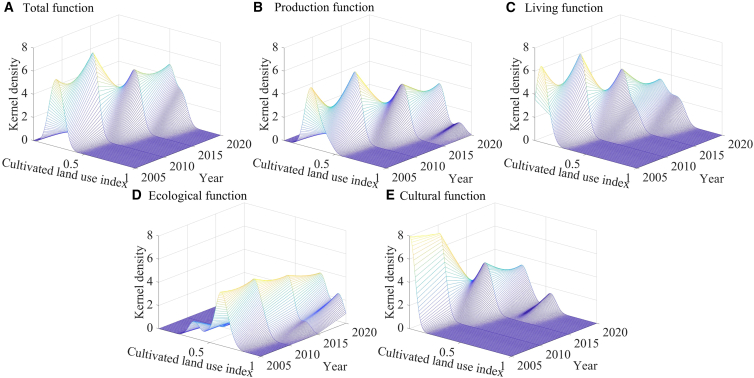
3D kernel density plot of recessive morphology of cultivated land use in the Ili River Valley, 2005-2020

During the study period, the dynamic characteristics of recessive morphology of cultivated land use are shown in Figure 5. To begin with, the center of the kernel density curve of the total function and sub-function has shifted to the right, indicating that the recessive level of cultivated land continued to improve. Among them, the rightward shift in the kernel density centers for cultural, living, production, and ecological functions gradually decreases in sequence. It indicates that the enhancement of cultural function has achieved significant improvement, while the growth trend of living and production functions has slowed down, and the ecological function has grown slowly. Additionally, the height of the kernel density curve of the total function and production function is relatively low, and the distribution pattern changes slowly. Their widths are widening and showing an upward trend. They show that the dispersion degrees are high and an upward trend, and the absolute difference continues to expand. Meanwhile, the waveforms of living, ecological, and cultural functions are different. Among them, the kernel density curve of living function has a moderate height and a gentle change, while its width is medium with a slight increase. It indicates that the dispersion degree is moderate and stable, with a moderate absolute difference that has slightly enlarged. The ecological function kernel density curve exhibits a high height with an upward trend, while its width is narrow and shows a downward trend. It indicates that the dispersion is low and shows a downward trend, with small absolute differences that continue to narrow. The height of the cultural function kernel density curve is low and remains stable for a long time, and its width is widened and stabilized. It shows that it has a high degree of dispersion, a great absolute difference, and long-term existence. Finally, the total function curve predominantly exhibited a single peak from 2005 to 2010, with a concentration in the low-value region. From 2010 to 2015, a high-value region emerged on the right side of the curve with an obvious peak, forming a clear double peak with the low-value region on the left, indicating significant polarization. From 2015 to 2020, the gap between the two peaks narrowed, and the polarization weakened. It shows that the degree of polarization increases and then decreases, the range of the high-value region expands continuously, and the low-value region gradually transitions to the middle-value region. The production function curve exhibits an overall fluctuating trend of “single peak concentration (2005), dual peak confrontation (2010–2015), and dual peak convergence (2020).” Among them, the phenomenon of local polarization was obvious in 2005–2015, and the high-value region gradually radiated to the low-value region. The living function curve is dominated by a single peak, but there is a small “shoulder peak” on the right side (high value region). In 2010, the living function exhibited local polarization, presenting overall weak yet stable local polarization characteristics. The ecological function curve is always dominated by a single peak, and the peak shape is sharp and concentrated in the mid-to-high value region, with extremely weak polarization characteristics. It indicates that the regional ecological functions are similar and there is no significant polarization phenomenon. The cultural function curve has always exhibited a bimodal shape, with local polarization from 2005 to 2015, and significant and stable overall polarization.

### Spatial patterns of recessive morphology of cultivated land use

**Figure 6:**
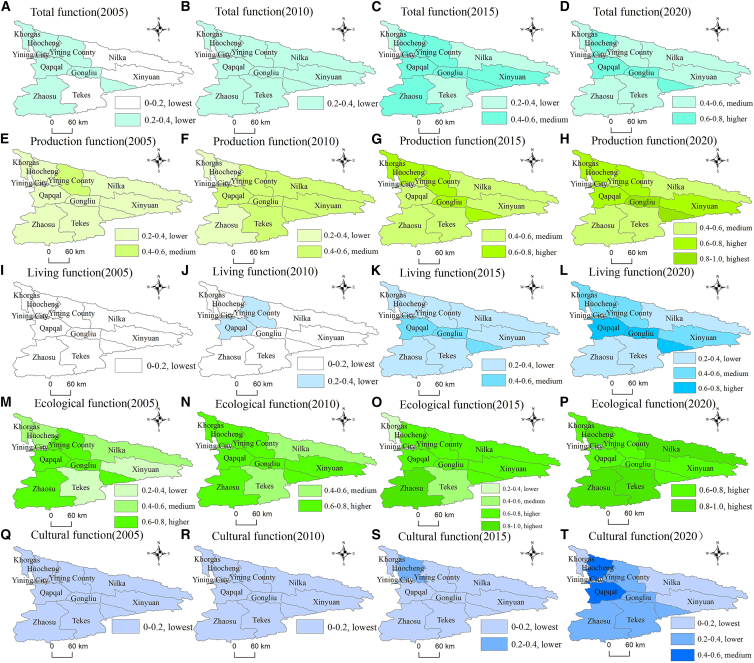
Spatial patterns of recessive morphology of cultivated land use in the Ili River Valley

The distribution pattern of recessive morphology of cultivated land use from 2005 to 2020 is shown in Figure 6. During the study period, the spatial pattern of the total functional index shifted from high in the west to low in the east to high in the river valley and low on both sides. In 2005, the trend of high in the west and low in the east was obvious. The western and eastern regions were respectively dominated by lower value regions and lowest value regions, with significant differences between the east and the west (Figure 6A). In 2010, the entire region exhibited lower values, with overall functional values not high, and no obvious differences between the north and south or east and west (Figure 6B). In 2015, it was mainly in the medium-value regions and widely distributed in the river valleys of the southeast, northwest, and southwest, while the lower value regions were scattered (Figure 6C). In 2020, the higher value regions concentrated in the river valley region in a northwest-southeast direction, while the medium-value regions were distributed on both sides of the river valley, with a clear trend of higher values in the river valley and lower values on both sides (Figure 6D).

The spatial differentiation of cultivated land production function in the Ili River Valley is obvious. In 2005, lower value regions were widely distributed throughout the region, and only Yining County was the middle-value region, with little regional spatial difference (Figure 6E). In 2010, there was a significant change in functional value levels (Figure 6F). Some local regions (Khorgas, Huocheng, Zhaosu, and Yining City) are still mainly lower value regions, while other counties and cities have risen to medium value regions and are concentrated in clusters in the central and eastern regions. In 2015, a pattern of high river valleys and low sides was formed (Figure 6G). The higher value region of the river valley shows a northwest-southeast direction and gradually weakens to the medium value region on both sides. In 2020, the situation of high river valleys and low sides remained the same (Figure 6H). The highest and higher value regions are concentrated in the central river valley region, forming a strip-like pattern. The medium value regions are primarily distributed in the southwest and northeast (including three administrative units: Zhaosu, Tekes, and Nilka).

The living function of cultivated land in the Ili River Valley gradually expanded from west to east. In 2005, the entire region was in the lowest value region, maintaining a low-value equilibrium state (Figure 6I). In 2010, the central part of the region (Qapqal and Yining County) was a lower value region, while the northwest, southwest, and east regions were all the lowest value regions (Figure 6J). In 2015, the river valley region in the central part was a medium-value region, with both its southern and northern sides being lower-value regions (Figure 6K). In 2020, the grade of the river valley region jumped to a higher value. The medium-value region was mainly distributed on the eastern side, encompassing six administrative units (accounting for 60%). And the lower-value regions are mainly located in the southwest and northeast (Figure 6L).

The ecological function of cultivated land presents a spatial pattern similar to its living function, gradually expanding from west to east. In 2005, the trend of high in the west and low in the east was obvious (Figure 6M). The western region is dominated by higher-value regions (except for some regions), while the eastern region is primarily composed of medium-value regions and lower-value regions. In 2010, the regions with higher values dominated, forming a northwest-southeast strip-like trend, and their area continued to expand. Only a few regions were medium value regions (Tekes and Nilka) (Figure 6N). In 2015, the region was mainly characterized by higher values, showing a northwest-southeast trend, with relatively small internal differences. Zhaosu in the southwest stands out as the highest-value region, with a few regions being medium-value regions (Tekes in the south) and lowest value regions (Khorgas in the northwest) (Figure 6O). In 2020, it presented a distribution pattern of low in the river valley and high on both sides (Figure 6P). The highest value regions are scattered on the northwest, southwest, and northeast sides, showing a “three pillars standing in a tripartite balance” pattern. And the higher value regions are distributed in a northwest-southeast strip pattern in the river valley area.

The spatial pattern of cultivated land cultural function has shifted from low-value equilibrium to high in the west and low in the east. In 2005 and 2010, the entire region consistently remained at a low level, with minimal spatial difference (Figures 6Q and 6R). In 2015, Huocheng in the northwest jumped to a lower value region, while the rest of the regions remained in a lowest value region (Figure 6S). In 2020, the trend was high in the west and low in the east (Figure 6T). The medium-value region was concentrated in the northwest (including Huocheng and Qapqal), the lower value region was distributed in clusters to the east and south of the medium-value region, and the lowest value region was distributed in the northwest and east of the region.

### Spatiotemporal differentiation in the coupling coordination degree of dominant and recessive morphologies of cultivated land use

#### Temporal changes in the coupling coordination degree of dominant and recessive morphologies of cultivated land use

**Figure 7:**
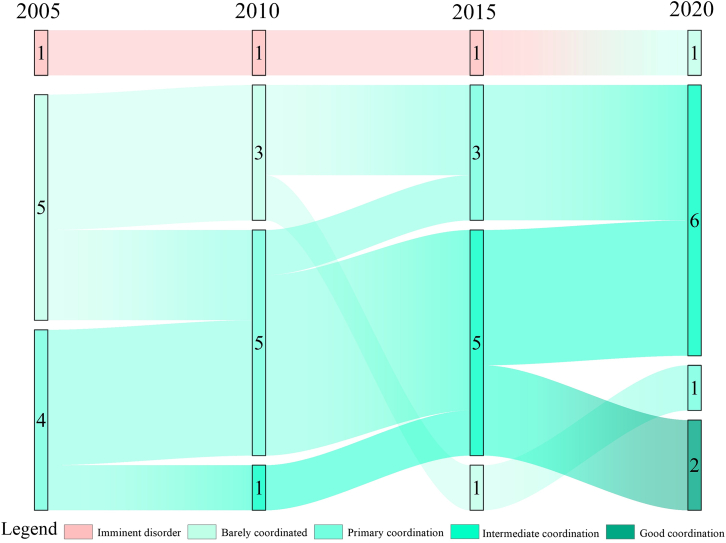
Temporal changes in the coupling coordination degree of dominant and recessive morphologies of cultivated land use in the Ili River Valley, 2005–2020 Note: The number in the figure indicates the quantity (in units) of counties and districts of this coupling coordination degree.

From 2005 to 2020, the coupling coordination degree of dominant and recessive morphologies of cultivated land use in the Ili River Valley can be divided into five categories: imminent disorder, barely coordinated, primary coordination, intermediate coordination, and good coordination (Figure 7). Overall, the coupling coordination degree increased from 0.5813 in 2005 to 0.7345 in 2020, indicating a transformation from barely coordinated to intermediate coordination. As of 2020, 80% of counties and districts have reached the stage of intermediate coordination and above. During the study period, the evolution of coupling coordination types of counties and districts in the region has shown varying degrees of improvement. It can be seen that the coordinated development of the region presents a good trend of continuous optimization and steady improvement, and the synergy of dominant and recessive morphologies of cultivated land use has significantly enhanced.

Between 2005 and 2015, although the type of imminent disorder persisted (one in number), the type of barely coordinated gradually decreased, the fluctuation of primary coordination types reduced, and the intermediate coordination continued to grow, indicating that the coupling relationship between regional systems continued to strengthen. This stage may have benefited from the in-depth implementation of the Western Development Strategy, which has facilitated the continuous improvement of infrastructure, effectively promoted the development of characteristic agriculture and animal husbandry as well as eco-tourism, and laid the foundation for the improvement of the coupling coordination degree. The persistent existence of the type of imminent disorder also indicates that the problem of regional development imbalance has not been alleviated. The possible reason is that due to the limitations of geographical location and the endowment of cultivated land resources, there is a certain imbalance between the development levels of dominant and recessive morphologies of cultivated land use. Between 2015 and 2020, the number of intermediate coordination types was the highest (increasing to six), and the good coordination type emerged (two in number), and the type of imminent disorder was completely eliminated. The possible reason is that, on the one hand, with the advancement of the Belt and Road Initiative, the region’s export-oriented economy has grown rapidly, leveraging the China-Kazakhstan Khorgas International Border Cooperation Center. On the other hand, after the 19th National Congress of the Communist Party of China’s proposal of the concept of high-quality development, regional development has prioritized ecological conservation, and has further advanced supply-side structural reform. The synergy between dominant and recessive morphologies of cultivated land use has significantly strengthened.

#### Spatial patterns in the coupling coordination stage of dominant and recessive morphologies of cultivated land use

**Figure 8:**
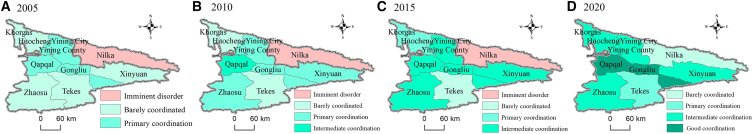
Spatial patterns in the coupling coordination stage of dominant and recessive morphologies of cultivated land use in the Ili River Valley, 2005-2020

Spatial visualization was conducted for the coupling coordination types of each county and district in the Ili River Valley in 2005, 2010, 2015, and 2020 (Figure 8). In 2005, the overall regional coupling coordination type was mainly characterized by barely coordinated and primary coordination, while only Nilka in the northeast was in a state of imminent disorder (Figure 8A). In 2010, the coupling coordination degree in the southern region improved (Figure 8B). Zhaosu and Xinyuan changed from barely coordinated to primary coordination, while Qapqal shifted from primary coordination to intermediate coordination. In 2015, the intermediate coordination expanded from Qapqal to surrounding counties and districts, spreading to Yining County, Gongliu, Xinyuan in the east, and Zhaosu in the southwest, covering a total of five administrative units, accounting for 50% (Figure 8C). In 2020, Nilka in the northeast and Tekes in the south transitioned to barely coordinated and primary coordination, respectively. Qapqal and Gongliu in the central part of the river valley were among the earliest to enter the stage of good coordination. The other counties and districts were all in the intermediate coordination stage (Figure 8D).

### Cultivated land use zoning and differentiated development strategies

**Figure 9:**
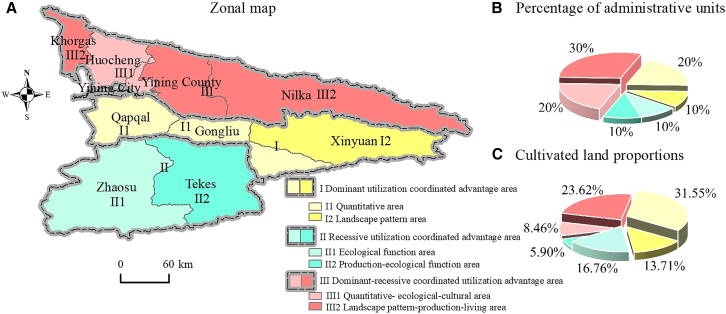
Zonal map of cultivated land use in the Ili River Valley

In view of the complexity of spatial differentiation of cultivated land use, based on the quantification of cultivated land use advantage index and coupling coordination degree, combined with the similarity of resource endowments and the integrity of administrative divisions, the region is divided into three primary and six secondary zones (Figure 9). In the primary zones, the proportions of administrative units in the dominant utilization coordinated advantage area (class I), recessive utilization coordinated advantage area (class II), and dominant-recessive coordinated utilization advantage area (class III) account for 30%, 20%, and 50%, respectively. The proportion of cultivated land area is 45.26%, 22.66%, and 32.08%, respectively. The administrative units and the proportion of cultivated land in various divisions of the secondary zones are shown in Figures 9B and 9C.

The characteristics and optimization recommendations for the primary zones are as follows.

1. Dominant utilization coordinated advantage area. It is concentrated in the central and eastern parts of the region, including three administrative units: Qapqal, Gongliu, and Xinyuan. The area of cultivated land is vast (accounting for 45.26%), with flat terrain, abundant water and heat, and a long history of agricultural reclamation. It is an important agricultural and pastoral production base, suitable for large-scale, intensive, and industrialized development. The beautiful ecological environment and unique farming culture have laid a solid foundation for the integrated development of eco-tourism and rural culture tourism. However, with the expansion of urbanization and industrialization, as well as the construction of infrastructure, a large amount of high-quality cultivated land has been occupied, and the problem of shrinking cultivated land area and deteriorating quality has become obvious. In addition, the outflow of a large number of young and high-quality labor forces has led to the aging of rural labor forces and the hollowing out of rural areas. In view of this, the future development direction of this area is as follows: (1) Actively promote the construction of high-standard farmland, improve infrastructure such as field roads and water conservancy pipe networks, and systematically restore the production potential of cultivated land. (2) Improve infrastructure and public services such as transportation and logistics, and achieve the coordinated development of cultivated land protection and urban-rural integration. (3) Cultivate “modern farmers” who master modern agricultural production and management techniques, promote the Internet and circular agriculture models, and facilitate the return of the labor force and the transformation of agriculture.
2. Recessive utilization coordinated advantage area. It is located in the southwest of the study area and includes two administrative units: Zhaosu and Tekes. This area is located in the upper reaches of the Ili River Valley, with abundant black calcareous soil and chestnut calcareous soil. The soil is fertile, and grasslands are widely distributed. It has a profound cultural heritage (such as the Tekes Bagua City and the Zhaosu Pastoral Care System), but it is fraught with crises such as the loss of inheritors and excessive commercialization. It is a national demonstration county for ecological civilization construction. However, unreasonable behaviors such as overgrazing and disorderly tourism development have weakened the ecological barrier function, and the contradiction between ecological protection and economic development is prominent. In view of this, the optimization suggestions in this area are as follows: (1) Promote the system of “rotational grazing + rest grazing + grazing prohibition,” improve the ecological monitoring and law enforcement system, and build a solid ecological barrier. (2) Organize activities such as intangible cultural heritage workshops and pastoral song singing to promote intergenerational cultural inheritance. Encourage herdsmen and intangible cultural heritage inheritors to cooperate in promotion to achieve the dynamic inheritance of core cultural resources. (3) Develop eco-tourism and rural tourism, strictly control the number of tourists and activities, promote themed and differentiated in-depth tourism activities to realize the deep integration of culture and tourism.
3. Dominant-recessive coordinated utilization advantage area. It is located in the northern part of the study area and includes five administrative units: Khorgas, Huocheng, Nilka, Yining City, and Yining County (accounting for half of the administrative region of the study area). Located in the core area of the horn-shaped opening of the Tianshan Mountains, it boasts abundant precipitation and fertile soil, offering significant advantages for agriculture, human settlement, and ecology. Primarily relying on traditional farming and primary agricultural product processing, it has not yet established a distinctive agricultural brand and faces a serious problem of industrial homogeneity. The location of the state capital and port boasts significant advantages in capital, technology, and talents, but transportation restricts the internal and external linkage. The optimization measures in this area are as follows: (1) Cooperate with local universities and research institutions to establish an agricultural science and technology innovation platform, focusing on tackling high-value-added technologies. (2) Promote the transformation of the agricultural economy from primary processing to the entire industry chain, including warehousing, packaging, logistics, and sales. (3) Fully explore the characteristic agricultural resources and products, build agricultural brands, apply for a number of agricultural products with geographical indications and green food, and promote the professional and characteristic development of the agricultural economy. (4) Upgrade the level of comprehensive transportation hubs, strengthen the construction of transportation infrastructure networks, and form a dual-wheel drive of domestic demand market and export-oriented economy.

The cultivated land use zoning and future development strategies are detailed in Table 1.

**Table 1:** Zoning and future development strategies of cultivated land use in the Ili River Valley

Primary zones	Secondary zones	Administrative region	Future development direction
I Dominant utilization coordinated advantage area	I1 Quantitative advantage area	Qapqal and Gongliu	advance the construction of high-standard farmland, deepen the strategy of “storing grain on the ground and storing grain in technology,” promote infrastructure construction, such as the renovation of farmland water conservancy facilities and land leveling, strengthen the quality of cultivated land, and improve production efficiency.
	I2 Landscape pattern advantage area	Xinyuan	focus on solving the problems of fragmentation of cultivated land and simplification of planting, optimize the structure of agriculture and animal husbandry and characteristic industrial clusters, in order to establish a modern agricultural industry system and achieve a large-scale and intensive cultivated land production layout.
II Recessive utilization coordinated advantage area	II1 Ecological function advantage area	Zhaosu	ensure pollution-free and efficient use of cultivated land, reduce agricultural non-point source pollution, improve agricultural resource utilization efficiency, promote coordinated economic and ecological development, and consolidate the foundation for green agricultural development.
	II2 Production-ecological function advantage area	Tekes	develop green high-value-added industrial clusters, vigorously promote ecological agriculture, facilitate industrial transformation, and enhance the production and ecological service functions of cultivated land.
III Dominant-recessive coordinated utilization advantage area	III1 Quantitative- ecological-cultural advantage area	Huocheng and Yining City	optimize planting structure, integrate advantageous agricultural products, promote ecological transformation and quality improvement of cultivated land, explore traditional farming culture, and build characteristic bases for ecological agriculture and farming civilization.
	III2 Landscape pattern-production-living advantage area	Khorgas, Nilka, and Yining County	repair the cultivated land basement, leverage the advantages of cultivated land aggregation and landscape diversity, promote the upgrading of agricultural mechanization and intelligence, build a high-yield and efficient agricultural industrial chain, and realize agricultural efficiency and farmers’ income increase.

## Discussion

### Analysis of influencing factors

This study finds that the dominant morphology of cultivated land use in the Ili River Valley has shown a slow trend of change since 2005. Among them, the quantity is growing slowly. The reason is that the Ili River Valley, as a typical intermountain basin terrain, features significant topographical variations within the valley and a large elevation difference. Moreover, the small area of regional valley plains limits significant fluctuations in cultivated land area, resulting in a slow growth trend in its quantity.^38^ Meanwhile, with the implementation of national ecological protection, food security strategy and high-standard farmland construction projects, the region strictly adheres to the red line for cultivated land protection, optimizes the layout of grain production, improves the quality and productivity of existing cultivated land, and restricts the disorderly expansion of cultivated land.^35^^,^^39^ Research indicates that the cultivated land landscape pattern index shows a slow downward trend, decreasing from 0.6366 in 2005 to 0.6194 in 2020, which reflects the decrease of cultivated land landscape dominance, connectivity, richness, and agglomeration (indicating an increase in fragmentation and more complex patch shapes). The possible reason is that with the upgrading of agricultural mechanization, specialization, and industrialization, the agricultural industrial structure has gradually developed to “unitization and large-scale,” and the cultivated land landscape has gradually shown a trend of homogenization.^40^ At the same time, the mountainous and river valley terrain results in cultivated land being characterized as “small and scattered.” Coupled with the demand for urban infrastructure, livelihood security, and ecological construction continues to grow, cultivated land is inevitably divided.^41^ As a result, the cultivated land landscape fragmentation has become increasingly prominent.^42^^,^^43^

Meanwhile, this study finds that the recessive morphology of cultivated land use (total function) in the Ili River Valley has shown a slow growth trend since 2005. Among them, the cultural function has seen rapid growth. The reason is that the Ili River Valley possesses the combined advantages of a beautiful ecological environment and a high concentration of ethnic minorities. The number of agricultural products with geographical indications and the area of agricultural tourism parks have increased year by year. Ethnic farming culture has been steadily developed, and the agricultural and cultural tourism industry has become an emerging pillar industry. At the same time, the living function has grown rapidly. The possible reason is that the region has leveraged national preferential policies for farmers to increase capital and technological investment, vigorously improve farmland infrastructure conditions, develop a characteristic industrial economy, promote the adjustment of rural industrial structure, and help farmers increase their income and become prosperous. Additionally, the production function has been growing slowly. On the one hand, the state attaches great importance to the protection of cultivated land and food security, and has issued a series of documents such as the “Cultivated Land Protection Law of the People’s Republic of China” and the “Food Security Assurance Law of the People’s Republic of China.” This has led to a steady increase in the amount of cultivated land and the gradual improvement in quality in the Ili River Valley. On the other hand, the average labor force bearing index rose from 0.5568 in 2005 to 0.8913 in 2020. This indicator shifted from a negative value to positive growth. This is contrary to the general trend of the rural population gradually shifting to urban areas as the socio-economic development progresses in China. It may be due to the high proportion of small-scale farmers operating in a decentralized manner. The inefficient extensive management mode constrains the rapid enhancement of cultivated land production function.^44^ Finally, the ecological function has grown slowly. The Ili River Valley, known as the “ecological barrier in the west,” has continuously promoted measures to reduce and optimize the use of chemical fertilizers and pesticides while exploring efficient irrigation models, resulting in an improvement in its ecological function.^45^ However, due to the scarcity of regional water resources and the dominance of traditional agriculture, the contradiction between increasing grain production and reducing agricultural non-point source pollution remains prominent, resulting in a relatively slow growth in ecological functions.^46^

### Findings and comparisons from the perspectives of dominant and recessive morphology

This study finds that the quantity of cultivated land use in the Ili River Valley shows a relatively stable development trend, a conclusion that has also been reached by existing literature.^47^ This is closely related to a series of territorial space planning measures and strict farmland protection policies formulated by China and local governments. Meanwhile, this study argues that all functions of cultivated land are showing an upward trend, albeit with varying degrees. Among them, the production and living functions increased greatly. The above conclusions are basically consistent with the research results of arid oasis region (Ganzhou District, Zhangye City),^48^ but contrary to the research conclusions of karst mountain area (Wulong, Chongqing).^49^ A possible reason is that both Ganzhou District in Zhangye City and the case of this study are located in the oasis plain region in the northwest, where agriculture is the dominant industry. With the continuous promotion of the national “Belt and Road” policy, along with the implementation of irrigation projects, water-saving technologies, the construction of agricultural industrial parks, and the training of “modern farmers,” it has promoted agricultural production and farmers' income.^50^ However, in the karst mountainous area of Wulong, Chongqing, there is a lack of surface water and infertile soil. Most of the agricultural products are for primary export and have a low processing and conversion rate, which makes farmers less enthusiastic about production.^51^ Simultaneously, the region is in a stage of rapid urbanization, with explosive growth in transportation, convenient internal and external connections, and a large number of young and middle-aged rural laborers turning to industrial and service sectors.^52^ It is worth noting that the cultural function of cultivated land in the case study area has grown explosively. Recent studies on Hebei province and the Min river basins respectively also agreed that the cultural function of regional cultivated land showed a continuous growth trend.^53^^,^^54^ It is the same as the supporting role of the evolution law of cultivated land functions revealed by diverse leisure demands driven by rapid economic and social development for regional sustainable development. The Ili River Valley, as a typical oasis agricultural area and a comprehensive tourism development zone in the arid region of northwest China, has increasingly highlighted its value in the development of agricultural cultural resources.^55^ It reflects China’s high regard for cultural functions during its high-quality development stage.

### Perspectives of the study

The contributions of this study are primarily manifested in three aspects: To begin with, the evaluation indicators are systematic, comprehensive, and scientific, covering both dominant and recessive attributes, which can provide scientific references for future research. The index system is further refined on the basis of the existing research.^17^^,^^56^ It not only includes dominant aspects such as Shannon’s diversity index and the aggregation index, but also innovatively incorporates recessive indicators such as the number of agricultural cultural heritage sites and the number of agricultural products with geographical indications. It makes up for the shortcomings of previous studies, which often lacked comprehensive coverage and emphasized material aspects while neglecting cultural ones. This provides valuable insights for the construction of the cultivated land use index system. Additionally, this study has achieved the cross-integration of multidisciplinary research methods, such as spatial mapping techniques in geography, landscape pattern models in ecology, and kernel density estimation in statistics. The research methods are scientific and reasonable, and they have overcome the limitations of existing research methods. This approach can not only comprehensively depict the spatiotemporal pattern of research results under multiple factors, but also provide methodological references and information support for similar studies. Finally, taking the Ili River Valley as a typical case, this study creatively combines the cultivated land use advantage index, coupling coordination degree, and regional realities to identify the competitive advantages among regional cultivated lands. It further proposes optimization strategies and development suggestions for cultivated land use, enhancing the practical rationality of the zoning results. It is helpful to the sustainable utilization of cultivated land and the rational planning of land space in arid oasis regions.

### Limitations of the study

In terms of functional zoning, A recent study used a clustering method based on the recessive morphology index and the coupling coordination degree to classify the study area into five types: H-H all advantages, H-H economic advantages, H-H ecological advantages, L-L economic advantages, and L-L none advantages.^10^ Not only did it identify spatial functional differences, but it also achieved the prediction of the transformation trajectory from 2021 to 2030 through ARIMA time series prediction and K-means dynamic clustering. In contrast, the zoning method based on the cultivated land use advantage index and coupling coordination degree in this study lacks the ability to diagnose the dynamic transformation path. Further research will further improve the scientific rigor and forward-looking nature of the zoning method. The aforementioned differences indicate that the regional cultivated land use zoning method may not be comprehensive enough. However, the research results can still reflect the future development direction and trend of cultivated land use in the Ili River Valley.

Factors influencing changes in cultivated land use are complex, diverse, and dynamic. Given the limited and difficult-to-obtain nature of data, this study has not quantified the extent, direction, and spatial heterogeneity of the effects of various influencing factors, nor has it delved into their deeper mechanisms of action. Therefore, in the future, it is necessary to further construct a scientific and reasonable indicator system to rationally quantify the influencing factors. By leveraging the geographically weighted regression (GWR) model to analyze the spatial differences of factors, exploring the complex driving mechanisms, deepening the research connotation of cultivated land use, and facilitating the optimal allocation of cultivated land resources.

It is particularly important to carry out empirical research on cultivated land use in ecologically fragile areas to verify and improve the theory, so as to alleviate the pressure of cultivated land use, ensure food security, and promote healthy and sustainable economic development. In this study, a two-dimensional index system of dominant and recessive morphologies of cultivated land use is constructed, and the differences in temporal and spatial patterns between dominant and recessive morphologies of cultivated land use in regional interaction are clearly considered. On this basis, by combining the dominant and recessive indices with the coupling coordination degree, the cultivated land use advantage index, and regional conditions, the competitive advantages of regional cultivated land are identified. It further proposes optimization strategies and differentiated development suggestions for cultivated land use, enhancing the practical rationality of the zoning results.

The following conclusions are drawn: (1) The dominant morphology of cultivated land use shows a slow growth trend, and its spatial characteristics have shifted from “medium values distributed in clustered patches” to “lower and medium values scattered on both sides of the river valley.” (2) The recessive morphology of cultivated land use (total function) exhibits a faster growth rate than that of the dominant morphology. At the same time, its spatial differentiation is obvious, shifting from higher in the west and lower in the east to higher in the central valley and lower on both sides. (3) The coupling coordination between dominant and recessive morphologies of cultivated land use has gradually shifted from barely coordinated and primary coordination to intermediate coordination. (4) The region is divided into three primary zones and six secondary zones, and this study proposes targeted differentiated development strategies. It is helpful to the sustainable utilization of cultivated land and the rational planning of land space in arid oasis regions.

From a theoretical perspective, the evaluation index system of this study innovatively incorporates recessive indicators such as the number of agricultural cultural heritage sites. It makes up for the shortcomings of previous studies, which often lacked comprehensive coverage and emphasized the material aspects while neglecting the cultural ones. Furthermore, by creatively combining the coupling coordination degree with the cultivated land use advantage index, this study has enriched the theoretical framework of cultivated land use research and further deepened the understanding of the differentiated development of dominant and recessive morphologies of regional cultivated land use. From a practical perspective, this study aims to provide a scientific basis for the conservation and utilization of cultivated land resources and the coordinated development of human-land relations, serving regional agricultural sustainability and comprehensive rural revitalization. Future research will further utilize the GWR model to analyze the spatial differences in influencing factors, in order to explore the understanding of the driving mechanisms of cultivated land use.

## Resource availability

### Lead contact

Further information and requests can be directed to the lead contact, Hui Xiang (xhui_123@163.com).

### Materials availability

This study did not generate new unique materials.

### Data and code availability

• This article analyzed publicly available data. These accession numbers for the datasets were listed in the key resources table.
• The article does not report any new code.
• Any additional information required to reanalyze the data reported in this article is available from the lead contact on request.

## Acknowledgments

We thank the staff members of government departments in Yining for their valuable suggestions and data support. We also acknowledge the financial assistance from our research group.

Funding was supported by the National Natural Science Foundation of China (42371288), 10.13039/100022820the Postgraduate Education and Teaching Reform Research Project in Yili Normal University (YSD2026JG10), the Youth Doctoral Program of “Tianchi Talents” in Xinjiang (2025QNBS010), and the Yili Normal University High-level Talent Project (2024RCYJ14).

## Author contributions

Conceptualization, C.Z., L.F., and H.X.; methodology, C.Z. and H.X.; investigation, C.Z.; writing – original draft, C.Z.; writing – review and editing, C.Z., L.F., and H.X.; funding acquisition, L.F. and H.X.; resources, C.Z., L.F., and H.X.; supervision, C.Z., L.F., and H.X.

## Declaration of interests

The authors declare no competing interests.

## STAR★Methods

### Key resources table

**Table:** 

REAGENT or RESOURCE	SOURCE	IDENTIFIER
**Deposited data**		

Land use data (Raster 30 m)	Chinese Academy of Sciences and the Geographic Spatial Data Cloud	http://www.resdc.cn/ https://www.gscloud.cn/
Statistical data (2005–2020)	Statistical Yearbook of Ili Kazakh Autonomous Prefecture and Xinjiang Statistical Yearbook	https://www.xjyl.gov.cn https://tjj.xinjiang.gov.cn
Other data	the National Landmark Query System and the Ministry of Agriculture and Rural Affairs of the People’s Republic of China	http://www.anluyun.com/ http://www.moa.gov.cn/

**Software and algorithms**		

ArcGIS 10.8	ESRI	https://www.esri.com
Fragstats 4.2	University of Massachusetts Amherst	https://www.fragstats.org
Origin 2024	OriginLab	https://www.originlab.com
MATLAB R2024b	MathWorks	https://www.mathworks.com

### Experimental model and study participant details

This study did not involve the use of animal models, human participants, plants, microbe strains, or cell lines. All analyses were conducted using the datasets described below and in the manuscript and therefore, no institutional oversight or ethical approval for animal or human subjects was required.

### Method details

#### Study area

The Ili River Valley is located in the western part of Xinjiang and the western section of the Tianshan Mountains, with a total land area of approximately 547,000 square kilometers. Of this, the cultivated land area is approximately 62,000 square kilometers, accounting for 11.42% of the total land area. The Ili River Valley belongs to temperate continental climate, with large temperature difference between day and night and sufficient light and heat. In addition, the terrain is a trumpet-shaped pattern of “surrounded by mountains on three sides” opening to the west, and the warm and humid air currents in the Atlantic Ocean drive straight in, with abundant precipitation, which is known as “the south of the Yangtze River beyond the Great Wall”. It has a flat and open terrain, wide distribution of black soil, deep and fertile soil layers, excellent agricultural production conditions and a long history of farming. It is a national key planting base. With the progressive implementation of the Belt and Road Initiative, the Western Development Strategy and the Rural Revitalization Strategy, the population of the Ili River Valley has continued to grow, human activities have increasingly impacted the land and the utilization of regional cultivated land has also undergone significant changes.

**Figure:**
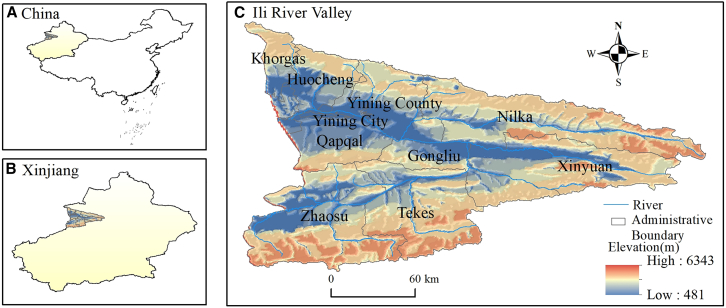
Regional profile of the study area

#### Data sources

Based on the availability and validity of the data, this study selected the data for the study area in 2005, 2010, 2015 and 2020.

**Table 2:** Data sources

Categories	Format	Sources	Data preprocessing
Land use data	Raster 30 m	Chinese Academy of Sciences (http://www.resdc.cn/) and the Geographic Spatial Data Cloud (https://www.gscloud.cn/)	landscape pattern data are mainly obtained by mask extraction and reclassification tools to acquire relevant indicator data.
Statistical data	/	Statistical Yearbook of Ili Kazakh Autonomous Prefecture (https://www.xjyl.gov.cn) and Xinjiang Statistical Yearbook (https://tjj.xinjiang.gov.cn)	the missing data of some years are filled by linear interpolation.
Other data	/	the National Landmark Query System (http://www.anluyun.com/) and the Ministry of Agriculture and Rural Affairs of the People’s Republic of China (http://www.moa.gov.cn/)	the missing data for some years are filled by linear interpolation.

Note: The city of Khorgas originally belonged to Huocheng County and was established in 2014. Therefore, before 2014, the data of Khorgas City and Huocheng County were consistent, but diverged after 2014.

#### Analytical framework

As the result of the interaction of natural environment and human activities, cultivated land use plays an important role in promoting socio-economic transformation and ecological sustainable development.^57^^,^^58^ Among these, the dominant morphology of cultivated land use changes includes quantitative characteristics and landscape fragmentation, while the recessive morphology of cultivated land use changes is dependent on dominant changes and primarily manifests in functional evolution. Currently, land use functions have evolved from the traditional three-dimensional (“production-living-ecological”) functions to a four-dimensional (“production-living-ecological-cultural”) framework. Therefore, this study explores the spatiotemporal pattern of cultivated land use in arid oasis regions from two aspects: dominant (quantitative and landscape pattern) and recessive (production-living-ecological-cultural) morphologies, taking the Ili River Valley as a case study and proposes optimization strategies. Finally, the number of regional cultivated land is stable, the structure is optimized and the function is improved.

**Figure:**
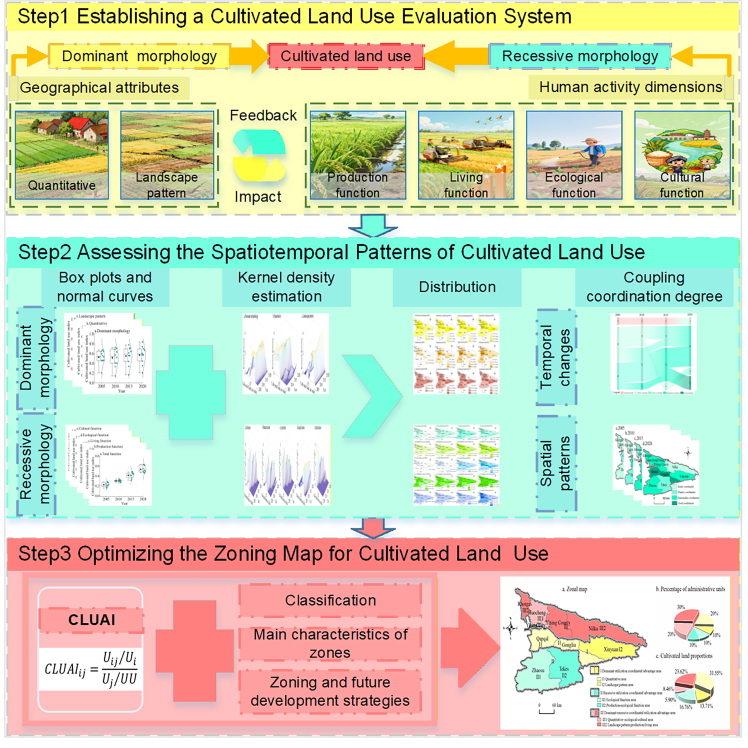
Theoretical framework of the study

#### Evaluation indicator system

Cultivated land is a regional complex with geographical environment attributes such as quantitative and landscape pattern, as well as human activity attributes such as production, living and ecological.^59^ With the transformation of urban and rural development and shifts in industrial structure, the dual dominant and recessive values of cultivated land use are increasingly prominent.^12^ From the perspective of dominant morphology of cultivated land use, quantitative characteristics are the basis of cultivated land use, which directly affects its outputs in terms of economic value, social security value, ecological value and other aspects. It is characterized by two indicators: rural per capita cultivated land area and cultivated land reclamation rate.^17^ The higher the indicator value in the range of cultivated land carrying capacity, the better the basis of cultivated land utilization and the more conducive it is to supporting the large-scale and specialized management of cultivated land in the quantitative level.^60^ Landscape pattern is the key support and important guarantee for the development of cultivated land resources, which is mainly manifested in the changes of cultivated land patches in fragmentation, connectivity, diversity and aggregation. Specifically, it is characterized by Patch Density (PD), Largest Patch Index (LPI), Landscape Shape Index (LSI), Patch Cohesion Index (COHESION), Shannon’s Diversity Index (SHDI) and Aggregation Index (AI).^61^^,^^62^ Among them, the higher the values of LPI, COHESION, SHDI and AI, the better the connectivity and spatial integration of the dominant patches in the cultivated land landscape, and the richer the landscape resources. The higher the values of PD and LSI, the higher the degree of cultivated land fragmentation and the more complex the patch shapes.

Meanwhile, the recessive morphology of cultivated land use is characterized by measuring the four functions of cultivated land: production, living, ecological and cultural. The production function mainly quantifies the utilization efficiency and output value of cultivated land through agricultural output.^63^ Good production function helps boost grain output and stabilize the willingness of agricultural workers to produce, thereby driving up the total agricultural output value. Therefore, the grain yield per unit cultivated land is used to measure the efficiency and value of the average labor force bearing index and per capita agricultural output value.^60^ The living function improves farmers’ employment security ability and increases their income levels by ensuring the food needs of urban and rural residents.^64^ Good living function helps to produce considerable grain output, promote the introduction of advanced production technology and improve hardware infrastructure. This will optimize the scale and structure of agricultural industry and meet farmers’ income expectations to a greater extent. Therefore, the per capita grain security rate is used to test the observability of grain output, while the level of agricultural mechanization and the farmer income intensity per unit of cultivated land are used to characterize the ability of cultivated land in providing employment security and livelihood support.^56^^,^^65^ Ecological function mainly measures the ecological supply capacity of cultivated land through various agricultural activities conducted by human beings and the background conditions of cultivated land. Good ecological function is mainly manifested in limiting the use of chemicals such as pesticides and fertilizers, and providing residents with green and organic ecological products. Thus, the fertilizer application intensity and the pesticide use intensity are used for characterization.^66^ At the same time, water resources are the primary factor limiting the development of arid oasis regions, so the water-saving irrigation area ratio is selected to evaluate the production and utilization mode of cultivated land in order to better realize the sustainable development of ecosystem.^67^ Cultural functions include the benefits of developing agricultural cultural resources and meeting the spiritual needs of urban residents.^68^ The development and utilization of agricultural cultural resources can enhance the popularity and influence of agricultural products, attracting more tourists to participate in activities such as enjoying rural scenery and experiencing agricultural life, thereby protecting, preserving and promoting agricultural culture.^69^ Therefore, the development of agricultural culture is discussed by using four indicators: the number of agricultural cultural heritage sites, the number of agricultural products with geographical indications, the area of agricultural tourism parks and the proportion of villages with rural characteristics cultural industry.^70^^,^^71^ Based on available data and an extensive literature review,^57^^,^^59^ a system of indicators was constructed from six dimensions: quantitative, landscape pattern, production, living, ecological and cultural.

**Table 3:** Evaluation index system for cultivated land use

Target layer	Factor layer	Indicator layer (Attributes)	Indicator calculation	Unit	Categories	Weight
Dominant morphology of cultivated land use	quantitative	X1 Rural per capita cultivated land area (+)	cultivated land area/rural population	hm^2^/person	statistical data	0.0485
		X2 Cultivated land reclamation rate (+)	cultivated land area/total land area	%	statistical data	0.0487
	landscape pattern	X3 Patch density (PD) (−)	see Shi et al., Ding et al.^61^^,^^62^	/	land use data	0.0501
		X4 Largest patch index (LPI) (+)	see Shi et al., Ding et al.^61^^,^^62^	/	land use data	0.0489
		X5 Landscape shape index (LSI) (−)	see Shi et al., Ding et al.^61^^,^^62^	/	land use data	0.0494
		X6 Patch cohesion index (COHESION) (+)	see Ding et al.^62^	/	land use data	0.0500
		X7 Shannon’s diversity index (SHDI) (+)	see Shi et al.^61^	/	land use data	0.0471
		X8 Aggregation index (AI) (+)	see Shi et al., Ding et al.^61^^,^^62^	/	land use data	0.0498
Recessive morphology of cultivated land use	production function	X9 Grain yield per unit of cultivated land (+)	grain production/cultivated land area	kg/hm^2^	statistical data	0.0498
		X10 Average labor force bearing index (−)	agricultural labor force/cultivated land area	person/hm^2^	statistical data	0.0504
		X11 Per capita agricultural output value (+)	gross agricultural output value/number of persons engaged in agriculture	yuan/person	statistical data	0.0479
	living function	X12 Per capita grain security rate (+)	total grain output/(permanent population ∗ 400 kg)	%	statistical data	0.0486
		X13 Agricultural mechanization levels (+)	total agricultural machinery power/number of persons engaged in agriculture	kW/person	statistical data	0.0475
		X14 Farmer income intensity per unit of cultivated land (+)	per capita net income of rural households/per capita cultivated land area in rural areas	yuan/hm^2^	statistical data	0.0478
	ecological function	X15 Fertilizer application intensity (−)	fertilizer application rate/cultivated land area	kg/hm^2^	statistical data	0.0506
		X16 Pesticide use intensity (−)	pesticide use rate/cultivated land area	kg/hm^2^	statistical data	0.0505
		X17 Water-saving irrigation area ratio (+)	water-saving irrigation area/total irrigation area	%	other data	0.0479
	cultural function	X18 Number of agricultural cultural heritage sites (+)	/	/	other data	0.0350
		X19 Number of agricultural products with geographical indications (+)	see Liu et al.^72^	/	other data	0.0442
		X20 Area of agricultural tourism parks (+)	/	hm^2^	other data	0.0464
		X21 Proportion of villages with rural characteristic cultural industries (+)	/	%	other data	0.0409

Note: “+” indicates a positive indicator, “-” indicates a negative indicator.

### Quantification and statistical analysis

#### Quantification of cultivated land use landscape pattern

Using Fragstats 4.2 software, the landscape pattern index is calculated to reveal the characteristics of cultivated land landscapes. Based on existing literature,^61^^,^^62^ six indicators are selected: Patch Density (PD), Largest Patch Index (LPI), Landscape Shape Index (LSI), Patch Cohesion Index (COHESION), Shannon’s Diversity Index (SHDI) and Aggregation Index (AI). The fragmentation, dominance, shape complexity, physical connectivity, richness and aggregation of cultivated land landscape patches were characterized, respectively.

#### Calculation of cultivated land use index

In order to eliminate the influence of different indicators due to dimensions and positive and negative differences and make the data comparable, it is necessary to perform standardization processing.^10^ The equation is:

(Equation 1) $$\begin{array}{c} Y_{ij}=\left\{ \begin{array}{c} \frac{X_{ij}-\min\left(X_{j}\right)}{\max\left(X_{j}\right)-\min\left(X_{j}\right)},the\;positive\;indicators\\ \frac{\max\left(X_{j}\right)-X_{ij}}{\max\left(X_{j}\right)-\min\left(X_{j}\right)},the\;negative\;indicators \end{array}\right. \end{array}$$

where *X*_*ij*_ and *Y*_*ij*_ represent the original value and standardized value of the j-th indicator of the i-th county or district respectively. *Max (X*_*j*_*)* and *min (X*_*j*_*)* denote the maximum and minimum values of the indicator.

The entropy weight method has the advantages of objectivity, scientificity and strong operability, and is the mainstream model for calculating weights.^10^ Therefore, the entropy weight method is used to calculate the weight of indicators. The equation is:

(Equation 2) $$\begin{array}{c} P_{ij}=\frac{Y_{ij}}{\sum_{n}^{i=1}Y_{ij}} \end{array}$$

(Equation 3) $$\begin{array}{c} E_{ij}=-\frac{1}{\ln\;n}\sum_{n}^{i=1}P_{ij}\;{\ln\;P}_{ij} \end{array}$$

(Equation 4) $$\begin{array}{c} U_{ij}=\frac{1-E_{ij}}{\sum_{n}^{j=1}\left(1-E_{ij}\right)} \end{array}$$

where *P*_*ij*_ represents the proportion of the standardized value of the indicator; *Y*_*ij*_ is the standardized indicator value; *n* is the number of evaluation indicators; *E*_*ij*_ stands for the entropy value of the j-th indicator for the i-th county or district, and *U*_*ij*_ represents the weight of the indicator.

#### Spatial mapping method

The spatial visualization of cultivated land use is conducted using ArcGIS 10.2. It can compensate for the limitations of traditional descriptive methods in paying attention to spatial concerns, making them suitable for spatiotemporal data undergoing multi-period dynamic evolution. In order to better reveal the temporal dynamics and spatial heterogeneity of geographical phenomena.^18^

#### Estimation of kernel density in cultivated land use

The kernel density estimation method is used to assess the temporal dynamic evolution of the kernel density of cultivated land utilization, in order to analyze its distribution position, morphology and polarization phenomenon. And Gaussian kernel density function is used to characterize its dynamic characteristics and development trend.^73^ It should be noted that the kernel density estimation method, as a distribution-free technique, has the advantage of intuitively presenting the probability distribution characteristics and multi-modal structure of the data, rather than conducting statistical inference tests.^74^ The equation is:

(Equation 5) $$\begin{array}{c} f\left(x\right)=\frac{1}{Nh}\sum_{i=1}^{N}K\left(\frac{x_{i}-x}{h}\right) \end{array}$$

(Equation 6) $$\begin{array}{c} K\left(x\right)=\frac{1}{\sqrt{2\pi }}e^{\left(-\frac{d^{2}}{2}\right)} \end{array}$$

where *f*(*x*) is the probability density of the cultivated land use index at attribute value *x*; *N* represents the total number of indicators; (*x*_*i*_-*x*) is the distance between the observed value of the i-th county or district and location x; *K(x)* is the Gaussian kernel function of cultivated land use index; *d* is the standardized distance; *h* is the bandwidth.

#### Coupling coordination degree of cultivated land use

The coupling coordination model is a mathematical model used to measure the interaction and coordinated development status between two or more systems. Based on existing research,^26^ this study employs explores the coupling relationship and coordination degree between the dominant and recessive morphologies of regional cultivated land use. The equation is:

(Equation 7) $$\begin{array}{c} C=\frac{{2\left(U_{1}\times U_{2}\right)}^{1/2}}{U_{1}+U_{2}} \end{array}$$

(Equation 8) $$\begin{array}{c} T=\alpha U_{1}+\beta U_{2} \end{array}$$

(Equation 9) $$\begin{array}{c} D=\sqrt{C\times T} \end{array}$$

where *C* represents the coupling degree, while *U*_*1*_ and *U*_*2*_ are the standardized values of dominant and recessive morphologies of cultivated land use respectively; *T* is the comprehensive coordination index, with a range of [0,1]. *α* and *β* are undetermined coefficients. Based on the concept of high-quality agricultural development and the government’s recent high attention for rural industrial revitalization, this study holds that the dominant and recessive morphologies of cultivated land use are equally important, both *α* and *β* are set to 0.5; *D* represents the coupling coordination degree, with a range of [0, 1]. The larger the value, the stronger the coupling coordination between the two systems. This study divides the coupling coordination degree (*D*) into 10 stages^26^.

**Table 4:** The division standard of coupling coordination stages

Disorder Recession Class		Coordinated Development Class	
*D*	coupling coordination stage	*D*	coupling coordination stage
0 ≤ *D* ≤ 0.1	extreme disorder	0.5 < *D* ≤ 0.6	barely coordinated
0.1 < *D* ≤ 0.2	severe disorder	0.6 < *D* ≤ 0.7	primary coordination
0.2 < *D* ≤ 0.3	moderate disorder	0.7 < *D* ≤ 0.8	intermediate coordination
0.3 < *D* ≤ 0.4	mild disorder	0.8 < *D* ≤ 0.9	good coordination
0.4 < *D* ≤ 0.5	imminent disorder	0.9 < *D* ≤ 1.0	high-quality coordination

#### Quantification of cultivated land use advantage

The Cultivated Land Use Advantage Index (*CLUAI*) is used to divide the advantage zones.^75^ The equation is:

(Equation 10) $$\begin{array}{c} {CLUAI}_{ij}=\frac{U_{ij}/U_{i}}{U_{j}/UU} \end{array}$$

where *CLUAI*_*ij*_ represents the advantage of cultivated land use of the j-th indicator in the i-th county or district. When *CLUAI*_*ij*_ > 1, it can be classified as an advantage area, with higher values signifying greater advantage and vice versa. *U*_*ij*_ represents the indicator value of the j-th indicator in the i-th county or district; *U*_*i*_ represents the average value of all categories of cultivated land use indicators in the i-th county or district; *U*_*j*_ represents the average value of cultivated land use of all j-th indicators; *UU* represents the average value of all indicators of cultivated land use in the study area.
